# PROTOCOL: Psychosocial interventions for preventing PTSD in children exposed to war and conflict‐related violence: A systematic review

**DOI:** 10.1002/cl2.1056

**Published:** 2019-10-10

**Authors:** Jennifer Hanratty, Laura Neeson, Tania Bosqui, Michael Duffy, Laura Dunne, Paul Connolly

**Affiliations:** ^1^ Campbell Collaboration UK & Ireland Queen's University Belfast Belfast Northern Ireland; ^2^ Centre for Evidence and Social Innovation Queen's University Belfast Belfast Northern Ireland; ^3^ Division of Social and Behavioral Sciences, Department of Psychology, Riad El‐Solh American University of Beirut Beirut Lebanon; ^4^ School of Social Sciences Education and Social Work Queen's University Belfast Belfast Northern Ireland

## 1 BACKGROUND

### 1.1 Description of the condition

It is estimated that one in 10 children (approximately 230 million children) currently live in a war or conflict‐affected society and will be exposed to daily violence in their communities (UNICEF, 2016). Some may be forced into violent combat, and many more will experience familial, social and cultural losses (Betancourt, McBain, Newnham & Brennan, 2013; Betancourt, Meyers‐Ohki, Charrow & Tol, 2013; IASC, 2014; Santa Barbara, 2006). It is generally accepted that children and young people exposed to violence in areas of conflict are at an increased risk of harmful effects, including injury, sexual abuse, disability, illness and long‐term mental health issues or psychological problems.

The harmful psychological effects of living through war or conflict include depression and anxiety disorders and post‐traumatic stress symptoms (PTSS) such as flashbacks, nightmares or intrusive thoughts about the trauma, avoidance of people, places or activities related to the trauma, disturbed sleep, disturbed play in young children and somatic symptoms (Attanayake et al., 2009; Dimitry, 2012; Fasfous, Peralta‐Ramírez & Pérez‐García, 2013; Jordans, Tol, Komproe & de Jong, 2009; Slone & Mann, 2016; Yule et al., 2000). While most people will experience some post‐traumatic stress symptoms following trauma, those whose symptoms persist and interfere with daily life may be diagnosed with post‐traumatic stress disorder (PTSD). A meta‐analysis of child and adolescent mental health in conflict affected settings estimated that prevalence rates were elevated for PTSD (47%, 17 studies, 95% CI: 35–60%), depression (43%, four studies, 95% CI: 31–55%) and anxiety (27% three studies, 95% CI: 21–33%) (Attanayake et al., 2009). This is compared to much lower lifetime prevalence estimates in the general population of, for example, American adolescents of 5% PTSD, 12% depressive disorder, 2.2% generalized anxiety disorder (Merikangas et al., 2010). A systematic review of the effect of war or conflict related violence on young children (age 0–6) found that prevalence of either PTSD or PTSS ranged from 8% to 45% (Slone & Mann, 2016). PTSD is the most common mental‐health condition associated with exposure to war, conflict or political violence (Attanayake et al., 2009; Betancourt, Borisova, et al., 2013; Dimitry, 2012; McDermott, Duffy, Percy, Fitzgerald & Cole, 2013; Slone & Mann, 2016).

As with adults, children suffering PTSD present with broad categories of post‐traumatic stress symptoms (re‐experiencing, avoidance/numbing and increased arousal). Younger children may display more overt aggression and destructiveness and re‐experiencing symptoms may take the form of re‐enacting the experience, repetitive play or frightening dreams. Subjective experience of the event and peri‐trauma factors, such as perceived severity and proximity, have been identified as possible risk factors for developing PTSD after trauma (Trickey, Siddaway, Meiser‐Stedman, Serpell & Field, 2012). Post‐trauma risk factors include low social support, poor family functioning (Trickey et al., 2012) and higher negative posttraumatic cognitions (Punamäki, Palosaari, Diab, Peltonen & Qouta, 2014). Finally, pre‐trauma factors, such as a non‐related mental health disorder, age and gender have also been linked to development of PTSD following war and conflict related violence. The exact nature of the relationship between age, gender and PTSD is unclear. There is some evidence suggesting that girls are at greater risk than boys because they may have higher levels of rumination or pre‐trauma anxiety (McDermott et al., 2013), girls may be more likely to experience greater subjective exposure than boys, but boys may exhibit more externalizing problems in response to trauma such as increased aggression (Dimitry, 2012). It may be that this different pattern of response in boys and girls reflects socially constructed gendered norms of appropriate behaviour and inequitable distribution of power and agency between boys and girls, but gender inequalities are understudied in the context of war and conflict and trauma more generally (Gilfus, 1999). Concerning age, older children are more likely to have direct exposure to conflict related violence (Dimitry, 2012) but younger children may be more vulnerable to developing PTSD as they lack the cognitive capability to process trauma that older children have developed. Others have argued that younger children may actually be protected by their less developed cognitive capacity as they cannot fully comprehend the meaning and implications of war and conflict (Barenbaum, Ruchkin & Schwab‐Stone, 2004).

In recent years there has been a noticeable shift in attention to the influence of mediating variables (e.g., cultural context, family/community support and personal capacity) and the importance of these influences in reducing the impact of war or conflict (Tol, Reis, Susanty & de Jong, 2010; Tol, Song & Jordans, 2013). This understanding has informed preventative psychosocial interventions, which aim to strengthen and improve protective factors for children living in war affected societies in order to inoculate children against the harmful effects of exposure to war, conflict or political violence. Having fundamental (although possibly relative) elements included in a intervention such as promoting community, self‐efficacy, a sense of hope, and feeling connected to a place may help reduce the negative effects of war (Betancourt, Borisova, et al., 2013; IASC, 2007).

While PTSD is common in children exposed to war and conflict related violence, it is important to note that not all children exposed to trauma will go on to develop PTSD. Severe distress and fear is a normal reaction to trauma and there is substantial natural recovery in the initial months and years after a traumatic event (Bisson et al., 2010). For example Punamäki et al. (2014) showed that 12% of children aged 10–12 exposed to war in Gaza suffered relatively low amount of post‐traumatic stress symptoms in the following year. A further 76% of children had initial high levels of symptoms but recovered within 11 months. A sizeable minority of 12% experienced initial severe levels of post‐traumatic stress symptoms which increased over a year. It is important to recognise that immediate intervention may not be necessary, and in the case of critical incident stress debriefing (CISD) may in fact be harmful (NICE, 2013; Rose, Bisson, Churchill & Wessely, 2002). Providing an intervention too early may interfere with the natural recovery process. A Cochrane Review of 11 trials involving adults indicated that CISD should not be routinely implemented with victims of trauma (Rose et al., 2002). The current evidence base for the use of debriefing with children is low quality (Pfefferbaum, Jacobs, Nitiéma & Everly, 2015; Jacobs & Pfefferbaum, 2015; Pfefferbaum et al., 2015) and while there is no current evidence of harm there is little empirical support for its use (Jacobs & Pfefferbaum, 2015; Pfefferbaum et al., 2015).

This raises important questions; when, if at all, should intervention be offered after a potentially traumatising event? How can we decide who does and does not need intervention to reduce the risk that PTSD will develop? Can at‐risk children be identified, screened and offered appropriate interventions?

## 2 DESCRIPTION OF THE INTERVENTION

This review focuses on psychosocial interventions that can be implemented with children following exposure to war and conflict‐related violence and will only include early interventions that aim to prevent childhood PTSD. We define psychosocial intervention as any intervention that offers psychological or social support (or both) with a goal of helping to prevent mental disorders developing (in particular PTSD) and improve long‐term mental health.

Universal interventions are offered to everyone in a population, regardless of the level of their exposure to war or conflict related violence. Selective interventions are targeted at subpopulations who may be at a higher risk of developing mental disorders, for example, only those directly exposed to war and conflict related violence. Indicated interventions are aimed at those already displaying some symptoms of disorder and who may benefit from intervention to prevent PTSD developing. We intend to include all three levels of intervention in this review.

The range of approaches that may be included in this review is broad. To illustrate the kinds of interventions that may be included we describe examples of potentially relevant interventions at each level.

### 2.1 Universal

Psychological First Aid (PFA), currently recommended by humanitarian guidelines (Sphere Project, 2004) to reduce distress after a humanitarian disaster through providing practical help, linking to services to meet basic needs for food, shelter and safety, along with listening and providing care and comfort.

### 2.2 Selective

A school based intervention that used mind‐body techniques to reduce PTSS among children in Gaza (Staples, Gordon & Abdel Atti, 2011).

### 2.3 Indicated

A classroom‐based intervention in Indonesia that focused on trauma processing and co‐operative play to reduce post‐traumatic stress symptoms and anxiety for children aged 8–12 affected by political violence (Tol, Komproe, Susanty, Jordans & De Jong, 2008).

The interventions described above are not an exhaustive list and we will include any interventions that provide psychological and/or social support to children affected by war and conflict that aims to prevent PTSD developing. Interventions may be delivered as a one‐off session, or over a number of weeks and they may be delivered by a trained professional or by a school‐teacher.

## 3 HOW THE INTERVENTION MIGHT WORK

Interventions to prevent the development of PTSD may work on a number of levels, from directly addressing and processing trauma through to improving individual, family or community resiliency and reducing distress for example, Jordans et al. (2010) and Khamis, Macy, and Coignez (2004). We do not yet claim to know the full universe of interventions that have been tested in this area and so, what follows, is a summary of the known mechanisms through which these interventions typically aim to bring about positive change. One goal of this review will be to examine intervention components to try to identify which components relate to greater effects.

### 3.1 Keeping children safe

By providing safe spaces for children to reduce the risk of further traumatisation and facilitate access to psychosocial support can reduce the risk of post‐traumatic symptoms developing into PTSD. For example.'Child Friendly Spaces' (CFS), primary goal is to protect children from further harm and traumatisation by reducing their exposure to potentially traumatic events, including the victimisation and abuse that children are at high risk of in emergency humanitarian settings. Child Friendly Spaces gives children spaces where they can play safely, whilst also creating opportunities to access psychosocial support and screening (Ager, Metzler, Vojta & Savage, 2013).

### 3.2 Community resiliency and capacity building

This focus on building community capacity through improved child protection to reduce risk of further trauma, and community level psycho‐educational/awareness raising activities and events to help communities support traumatised children through greater understanding of normal reactions to trauma. Community level psychosocial interventions can encourage groups of participants to reflect on difficult times and aim to develop coping skills to allow them to face trauma‐related experiences in a supportive social environment for example, Kumakech, Cantor‐Graae, Maling, and Bajunirwe (2009). Peltonen and Palosaari (2013) connect the benefits of resiliency interventions on short‐term impacts (e.g., reducing the likelihood of trauma‐related symptoms) and long‐term impacts (i.e., the child has a resource to draw upon for life), with improved relationships with their family and connectedness to their community. Community level interventions can also help to reduce stigma (Betancourt, Agnew‐Blais, Gilman, Williams & Ellis, 2010).

### 3.3 Social and community connectedness

Many psychosocial support programmes include a community component in which children's engagement in their local community is thought to increase hope, social connectedness and prosocial behaviour, and to reduce externalising symptoms such as aggression by increasing awareness of, and attachment to, the wider social environment. Activities may include community events, volunteer work or public theatre (Constandinides, Kamens, Marshoud & Flefel, 2011).

### 3.4 Supporting family structures

The literature suggests that, in order to be able to withstand the harmful effects of living in a conflict‐affected society, it is vital for children to have loving, secure and consistent relationships with their caregivers (Betancourt, Borisova, et al., 2013; Qouta, Punamäki & El Sarraj, 2008; Thabet, Ibraheem, Shivram, Winter & Vostanis, 2009). Caregiver‐focused support intervention*s* aim to protect dependents from the adverse consequences of experiencing conflict‐related harm by improving family structures, for example, improving the relationship between parent and child, increasing parental involvement and reducing the risk of parental stress (see Dybdahl, 2001). This may include improved parenting skills, improved attachment behaviours or parental psycho‐education, all of which aim to assist parents in meeting the needs of their children and promoting their well‐being. Psycho‐education for example may work by helping parents to understand the symptoms of PTSD, how this may manifest in a child and how parents can best support their child after exposure to a traumatic event.

### 3.5 Peer group support

Therapies based in groups, often within a psycho‐educational or skills based therapeutic model, draw on an added mechanism of change by drawing on peer influence and support. Group settings are used to normalise experiences, to alleviate shame, to build cooperative behaviours and provide a forum to practice skills (Bolton et al., 2007). Classroom based interventions use much the same rationale, but with the addition of a real world setting to further normalise and integrate learning (Constandinides et al., 2011). These formats are also used given the larger number of beneficiaries that can be reached with few resources (O'Callaghan, McMullen, Shannon, Rafferty & Black, 2013).

### 3.6 Individual psycho‐education and skills teaching

Interventions that use psycho‐educational techniques endeavour to use education, information and insight to protect and promote well‐being and to challenge misperceptions and taboos. Providing evidence‐based information on traumatic reactions and living through the daily stressors of war is thought to help normalise experiences, to screen for more serious reactive disorders and encourage healthy and adaptive coping responses (Betancourt, Meyers‐Ohki, et al., 2013). For example, Individual Psychological First Aid aims to strengthen mental health outcomes immediately after conflict by providing psycho‐education on posttraumatic reactions and encouraging positive coping strategies in the immediate aftermath of the potentially traumatic events(s) (Betancourt, Meyers‐Ohki, et al., 2013).

### 3.7 Emotional regulation

Many psychosocial preventative interventions include teaching the ability to self‐regulate emotions during or after a traumatic event occurring using, for example, breathing exercises, help seeking, social connectedness or positive self‐talk. For example, Punamäki et al. (2014) evaluated the effectiveness of the psychosocial intervention 'Teaching Recovery Techniques' which is based on cognitive behavioural therapy (CBT) principles and provides several ways of increasing emotion regulation, expression and recognition. This in turn can help children to develop effective coping skills, to feel empowered and be able to regulate their emotions using narrative, imagery and psychoeducational techniques.

### 3.8 Trauma processing

Psychosocial preventative interventions that incorporate trauma processing techniques aim to facilitate the integration of traumatic memories into autobiographical memory in order to reduce PTSS and the risk of PTSD. Trauma processing is most commonly used to treat PTSD through narrative storytelling, such as in KidNET (Neuner et al., 2008) or imaginal and in vivo exposure to specific distressing, and often intrusive, memories, such as in Trauma‐Focused CBT (TF‐CBT; Brown et al., 2017). Some interventions have incorporated these techniques for children with PTSS as part of a wider aim of healing, through play and guided imagery (Peltonen & Punamäki, 2010), to help to integrate and assign meaning to traumatic experiences (Apfel & Simon, 1996) and for indicated secondary prevention interventions for children already displaying symptoms of PTSD (Tol et al., 2008).

### 3.9 Cognitive restructuring

Some trauma focused interventions, usually derived from CBT, include the identification and evaluation of unhelpful thoughts and appraisals of traumatic experiences (such as self‐blame) in order to help integrate fragmented and intrusive thoughts about traumatic experiences (Peltonen & Punamäki, 2010).

## 4 WHY IT IS IMPORTANT TO DO THIS REVIEW

Children living in areas of conflict are at elevated risk of negative mental, emotional and behavioural outcomes, including high rates of PTSS, PTSD and depression and anxiety problems (Attanayake et al., 2009; Dimitry, 2012). There are multiple studies on the immediate impact or war and conflict‐related violence on children but few studies on the long‐term impacts (Attanayake et al., 2009; Shaw, 2003), or on the impact psychosocial preventative interventions can have. Few reviews explicitly address the mechanisms of change (Betancourt, Borisova, et al., 2013; Brown et al., 2017; Peltonen & Punamäki, 2010).

There is professional debate around which approach is most effective and least harmful to children living in war or conflict‐affected societies, and whether only children with a diagnosed condition should be treated (Apfel & Simon, 1996; Betancourt, Borisova, et al., 2013). The inevitable fact of limited resources in these contexts may mean resources are directed to those who are perceived to be in most immediate need, at the expense of 'inoculating' all children from potential future problems. What is vital, as we have learned from the debriefing trials in adults, is that interventions should not be harmful or inadvertently disable adaptive responses to trauma. It is currently recommended that children exposed to war or conflict‐related violence should not be given pharmacological intervention (IASC, 2007, 2014), so psychosocial interventions provide an important alternative response to try to prevent mental health problems developing.

Not all children exposed to trauma will go on to develop PTSD but it is assumed what works for adults will work for children as well. However, there is no evidence to support this assumption and less still is known in what is effective to prevent PTSD for children aged under 11. Adults and adolescents can have broadly the same techniques applied in interventions but this does not apply so readily to children where there is a much larger gap and less clear guidance around what works. There is the potential for vast clinical relevance if we can understand more in this area, especially in the context of childhood PTSD and exposure to war and conflict‐related violence. We don't yet know which children are most at risk of developing PTSD following trauma and which children are likely to recover without intervention. Nor do we know how best to screen for potential problems and who to offer intervention too as a result. One aim of this review will be to examine which children are most likely to benefit from intervention.

### 4.1 Existing reviews

There are a number of relevant existing reviews detailed below. Our review and existing/ongoing reviews differ in three ways. First we will not be limiting our inclusion criteria to studies conducted in low income countries. Second, we will not be limiting our review to randomised controlled trials. Finally, our review will focus on prevention of PTSD rather than treatment.

Reviews which focus only on LMIC's (e.g., Purgato et al., 2015, 2016, 2014; Morina, Malek, Nickerson & Bryant, 2017; Brown et al, 2017) overlook interventions for trauma‐exposed youth in high income countries (HIC), such as Northern Ireland, Cyprus and Israel.

Reviews that only include RCTs (e.g., Purgato et al., 2015, 2016, 2014; Morina et al., 2017; O'Sullivan, Bosqui & Shannon, 2016; Brown et al., 2017) risk missing interventions developed and evaluated within the LMIC context. The well conducted trials tend to be based on interventions developed in the west and so by including only RCTs 'home grown' interventions may be excluded. This means they may not capture the interventions being delivered in the challenging context of ongoing conflict and violence with limited resources which precludes the use of RCT designs, for example studies like (Ager et al. (2011), Jordans, Tol, Ndayisaba, and Komproe (2013), Loughry et al. (2006); Thabet, Vostanis, and Karim, (2005)).

As our review is focused on prevention of PTSD rather than treatment, treatments have been evaluated by existing reviews (Purgato et al., 2015; Morina et al., 2017).

Other reviews have provided useful narrative summaries of existing interventions but have been limited in their search dates (Barry, Clarke, Jenkins & Patel, 2013; Betancourt, Borisova, et al., 2013; Forman‐Hoffman et al., 2013; Jordans et al., 2009; Jordans, Pigott & Tol, 2016; Peltonen & Punamäki, 2010), did not focus on children in war‐ or conflict‐affected settings (Barry et al., 2013; Blom & Beltran, 2010; Ehntholt & Yule, 2006; Gillies, Taylor, Gray, O'Brien & D'Abrew, 2013) or were not systematic reviews (Blom & Beltran, 2010; Ehntholt & Yule, 2006).

Countries affected by war and other crises may not have the resources or be in the position to offer the stable and secure environment needed to achieve the acceptable level of rigour in an RCT. For these reasons, we intend to include RCTs, quasi‐randomised controlled studies and non‐randomised studies (NRS) to allow for the possibility of including more culturally ‐ and context ‐ specific interventions developed within LMIC, whilst not excluding HIC from our review. Given the number and scale of ongoing conflicts worldwide and the huge numbers of children affected by war and conflict related violence, this review is likely to be useful to policy makers and service commissioners around the world and aid organisations working in conflict affected countries.

### 4.2 Objectives

To assess the effectiveness of psychosocial interventions for preventing PTSD in young children aged 0–11 years old living in war and conflict‐affected societies.

### 4.3 Methodology

#### 4.3.1 Criteria for including and excluding studies

##### Types of study designs

Randomised controlled trials (RCTs) and quasi‐RCTs (where participants are allocated to groups using a quasi‐random method such as date of birth or alternate allocation). Quasi‐experimental controlled studies with non‐random allocation, that is, non‐randomised studies (NRS), in which researchers have prospectively allocated participants (or settings, clinics, locations, etc.) to intervention or control conditions we be included.

We will not include studies with historical controls, case control studies, cross‐sectional studies or case reports/case series.

##### Types of participants

Children aged from birth to 11 years who have been exposed to war and conflict‐related violence. Studies including children older than 11 years can be included providing that the mean age of participants is under 12. In the case of studies where the mean age of participants is older than 12, study authors will be contacted to request summary data to calculate effect sizes for children under 12.

We are interested in that population which does not have a formal diagnosis of PTSD. As PTSD is the most commonly diagnosed mental health condition we are interested in interventions which aim to protect and support young people from developing this disorder, rather than those which are delivered, as treatment, to those with a formal diagnosis of PTSD. Therefore, we will exclude studies targeting children who have received a formal diagnosis of chronic PTSD. In studies with a mixed population (those with and without formal diagnosis), or where PTSD diagnosis was not assessed as baseline, we will include studies where the majority of participants have not been identified as meeting the criteria for chronic PTSD and where it is clear that the intervention is aimed at preventing PTSD developing into a chronic condition.

We will also exclude children who are refugees or asylum seekers unless they are internally displaced, or displaced to another location that remains affected by war and conflict‐related violence. This is because we feel refugee children and asylum seekers will have additional issues due to migration stressors and we want to focus on interventions for children who are still living in conflict settings or living with the aftermath of conflict.

##### Types of interventions

Any psychosocial intervention that has an element of preventing PTSD or reducing PTSS and is delivered in any conflict‐affected setting (e.g., school, home or community), to children or their caregivers, compared with no intervention, waitlist control, treatment as usual or any other inactive control condition. We define psychosocial interventions in this context/setting as any intervention that offers psychological or social support (or both) with a goal of helping to prevent mental disorders developing (in particular PTSD) and improve long‐term mental health.

Interventions aimed at children affected by one‐off acts of terrorism (e.g., 9/11) or a natural disaster will be excluded as this population are, in general, not living under threat of continued violence, war or conflict. We will exclude studies that include children with a diagnosis of chronic PTSD. We will also exclude interventions whose main focus is to treat PTSD rather than prevent it.

Interventions which were originally designed to treat interpersonal traumas (e.g., TF‐CBT) may be included so long as the intervention is being used to prevent PTSD and/or reduce PTSS.

##### Types of outcome measures

We will only accept standardised measures, their culturally specific equivalents, or both.

##### Primary outcomes

1. *PTSD diagnosis.
2. *Acute stress reactions and post‐traumatic stress symptoms. Examples of measures include the Child Post‐traumatic Stress Scale (CPSS; Foa, Johnson, Feeny & Treadwell, 2001), the Child War Trauma Questionnaire (CWTQ; Macksoud, 1992), and the Acute Stress Checklist for Children (ASC‐Kids; Kassam‐Adams, 2006).
3. *Adverse effects/outcomes for example stigmatisation, reluctance to seek future treatment, worsening of symptoms/problems.

##### Secondary outcomes

4. Resilience indicators for example hope or pro‐social behaviour. Examples of measures include the Children's Hope Scale (CHS; Snyder et al., 1997), the SDQ (Goodman, 2001) or the Emotional Regulation Questionnaire for Children (Gullone & Taffe, 2012).
5. Social relationships for example family relationship, peer relationships and attachment security). Examples of measures include the 10‐item Security Scale (Kerns, Klepac & Cole, 1996) which measures children's attachment to parents.
6. *Internalizing symptoms as a potential overlapping traumatic reaction for example anxiety, depression and culturally specific equivalents. An example measure is the Screen for Child Anxiety Related Disorders, 5‐item version (SCARED‐5; Birmaher et al., 1999).
7. Externalizing symptoms as a potential overlapping traumatic reaction for example aggression, conduct problems, anti‐social behaviour and culturally specific equivalents. Example measures include the Child Behaviour Checklist (CBCL; Achenbach and Rescorla, 2000, 2001), the Strengths and Difficulties Questionnaire (SDQ; Goodman, 2001), and the African Youth Psychological Assessment (AYPA; Betancourt, Yang, Bolton, and Normand, 2014).

*We will use those outcomes marked with an asterisk (*) to populate the 'Summary of findings' table. If data are insufficient, we will provide a narrative account of the outcomes.

##### Duration of follow‐up

We will include all follow‐up data in included studies. For synthesis follow‐up periods will be grouped into categories of immediate post‐intervention (0–1 month), short term follow up (1–3 months), medium term follow up (3–12 months) and long term (more than 12 months post‐intervention).

##### Types of settings

We will include any psychosocial intervention that has an element of reducing PTSS or preventing PTSD and is delivered in any war‐affected setting (e.g., school, home or community), to children or their caregivers.

#### 4.3.2 Search strategy

The search strategy was developed by extracting the search terms from previous relevant reviews and the keywords from a random selection of five relevant published trials. The terms were categorised into those relating to the participants, setting and intervention. Each term was then searched for in the database thesauri in OVID Medline and a complete list of potentially relevant terms constructed. Review authors reviewed each list and added any missing terms and removed redundant terms. The search strategy was then tested to ensure that eligible studies already identified were found.

The search strategy includes search strings for participants (children), setting (war/conflict, political violence) and intervention (psychosocial). We will use the search strategy in Appendix A to search OVID Medline and adapt for the databases listed below. Where possible database specific limiters will be applied (e.g., the use of verified age limiters) to limit the number of irrelevant search results.

We will search the following and we will not apply any date, language or publication status restrictions;

##### Databases

- Cochrane Central Register of Controlled Trials (CENTRAL), part of The Cochrane Library, which includes the specialised register of the Cochrane Developmental, Psychosocial and Learning Problems Group. (All years).
- Published International Literature on Traumatic Stress (PILOTS, all available years).
- PsycINFO (1806 to current; OVID).
- EMBASE (1974 to current; OVID).
- Ovid MEDLINE (1946 to current, including In‐Process and Other Non‐Indexed Citations).
- CINAHL Plus (1937 to current; EBSCOhost).
- Science Citation Index (1970 to current; Web of Science).
- Social Sciences Citation Index (SSCI; 1970 to current; Web of Science).
- Conference Proceedings Citation Index ‐ Science (CPCI‐S; 1990 to current; Web of Science).
- ProQuest Dissertations & Theses; UK & Ireland (for dissertations).
- WorldCAT (limited to theses) worldcat.org/
- Google Scholar.

##### Grey literature

- International Initiative for Impact Evaluation (3ie) ‐ http://www.3ieimpact.org/
- UNICEF Evaluation and Research Database ‐ https://www.unicef.org/evaldatabase/
- World Health Organisation Mental Health in Emergencies http://www.who.int/mental_health/emergencies/en/
- Children and Armed Conflict Unit https://www1.essex.ac.uk/armedcon/themes/psychosocial/default.htm
- War Child https://www.warchild.org.uk/
- DFID Research for development https://www.warchild.org.uk/
- International Rescue Committee http://oef.rescue.org/#/?_k=pn0gi6
- The Mental Health and Psychosocial Network https://mhpss.net/
- USAID https://www.usaid.gov/
- The European Commission's Humanitarian Aid and Civil Protection http://ec.europa.eu/echo/index_en

##### Clinical trials registers

- World Health Organisation International Clinical Trials Registry Platform (ICTRP; http://apps.who.int/trialsearch; all available years).
- ClinicalTrials.gov (http://clinicaltrials.gov; all available years).
- ISRCTN (ISRCTN.com; all available years).

##### Search for systematic reviews

- The Campbell Collaboration Library of Systematic Reviews (campbellcollaboration.org/lib/; current issue).
- Cochrane Database of Systematic Reviews (CDSR; current issue; *The Cochrane Library*).

##### Searching other resources

In addition we will search the reference list of all included studies and existing relevant reviews to identify other relevant studies. We will also contact authors of included studies and experts in the field about any relevant unpublished trials. Finally, we will contact leading charitable and non‐governmental organisations to obtain reports/service evaluations of any relevant interventions including; Save the children, War Child, Oxfam, MSF, The Red Cross, Catholic Agency For Overseas Development (CAFOD), Medicins du Monde.

We will also include a series of google searches using the search strings and scanning the titles of the first 2 pages of each search.

##### Selection of studies

One author will remove duplicates and obviously irrelevant records based on a preliminary screen of titles. Two reviewers will then screen the title and abstract of a random selection of 10% of the remaining records independently and discuss decisions. This is to ensure consistency in application of eligibility criteria. Subsequently, records will be screened by one reviewer only and supported by text mining classification. An additional random selection of 10% of records will also be coded by at least two reviewers to ensure consistency in decision making throughout the screening process. Full texts of potentially relevant studies will be retrieved and two review authors (LN and one other) will independently assess full‐text articles against selection criteria. Any disagreements will be resolved through discussion with the author team. Reasons for exclusion will be recorded and presented in the 'Characteristics of excluded studies' table for records excluded at the full text stage.

##### Data extraction and management

Two review authors will independently extract data using a standard extraction form using DistillerSR software. Any disagreements will be discussed until a consensus is reached.

#### 4.3.3 Description of methods used in primary research

Primary studies eligible for inclusion must prospectively compare a group receiving a relevant intervention to another group, not receiving the intervention. Some studies will be randomised controlled trials, where participants are randomly assigned to intervention or control groups. Other studies may employ a quasi‐randomised controlled approach, where participants are allocated to groups using a non‐random method (date of birth, alternate allocation). We will also include non‐randomised studies (NRS) where researchers have prospectively allocated participants (or settings, clinics, locations, etc.) to intervention or control conditions and taken steps to avoid or control for potential confounds. Steps may include matching participants, testing to ensure baseline equivalence of intervention and control groups on key measures, using pre‐ and post‐test comparisons or change from baseline, using multilevel models controlling for potential confounders, appropriate adjustments to account for clustering where relevant.

We anticipate that studies will use a range of approaches and informants when measuring outcomes, from clinical diagnostic tools to participant self‐reported symptoms or parent/teacher ratings. Careful consideration will be given to which means of measuring the same outcome can be reasonably combined in meta‐analysis. For example parent and teacher reported outcomes, child self‐reports and professional diagnosis may produce consistently different estimates of effect. Sensitivity analysis will be carried out to ascertain the impact of these decisions.

We will not include studies with historical controls, case control studies, cross‐sectional studies or case reports/case series.

#### 4.3.4 Criteria for determination of independent findings

##### Multiple reports of a single study

We will treat multiple reports generated by a single study as one study and extract data accordingly. Where it is unclear if two or more reports refer to the same study we will discuss as a group and consult with report authors for confirmation.

##### Multiple groups in a single study

In our analysis we will combine data from all eligible intervention arms and compare these with all eligible control arms, resulting in a single pairwise comparison. If this is not advisable, for example where a study compares two distinct intervention approaches with one control group, then we will analyse each intervention group separately and divide the sample sizes for the comparison/control group by the number of intervention arms to avoid double‐counting (and vice‐versa if there is one intervention compared to multiple similar control groups). The exception to this would be the case where a study reports multiple effect size estimates that are truly independent of each other, for example reporting separately on children under and over a certain age or boys and girls separately. If the effect size estimates are truly independent they will be included as separate estimates of effect.

##### Multiple measures of the same construct

If studies use multiple measures to assess the same construct at the same time point we will select the outcome measure common to the majority of studies. Where no common measures are used across studies we will calculate the mean SMD across all measures of the same construct within each study, together with their pooled estimated variance so that each study contributes only one effect estimate to any meta‐analysis.

##### Multiple measures of the same outcome

In the case of multiple informants for the same outcome, for example child, parent and teacher report, we will consider averaging across all informants or conducting separate analysis for each informant. The decision to combine data from multiple informants or keep them separate will be based on consideration of the correlation between measures from different informants (if reported by study authors) and sensitivity analysis with tests for subgroup differences to assess the convergence/divergence between each informant.

##### Multiple points in time

If outcome data are reported at different time‐points we will perform separate analysis on post‐intervention outcome data according to the following time frames: up to one month (post intervention), one to three months (short term), six to 12 months (medium term) and 12 months or more post intervention (long term). If any study reports on two timepoints falling into one of the above time‐frames we will calculate an average effect estimate for each timeframe.

#### 4.3.5 Details of study coding categories

The data extraction and coding form has been devised and piloted by the authors (see Appendix A). Briefly studies will be coded and data extracted as follows:

- General information; study author, date, location, source, unique report ID, study ID (if multiple reports of the same study are included), author contact details.
- Contextual information; Funding source, if the intervention is research or practice led, if the intervention is developed within or without the cultural context in which it is delivered, information relating to the nature and duration of the conflict or war.
- Participants' information; age, gender, any selection criteria applied, type of traumatic event, reasons for recruitment or referral to the study, attrition rates and reasons given.
- Intervention and control characteristics; intervention aims, components of the intervention, who is involved (i.e. children only or parents, teachers etc), how long after the event is the intervention delivered, where the intervention is delivered, conflict or post‐conflict setting, training received by providers, provider qualifications, number and duration of sessions, manualised delivery or not, target of the intervention (universal, selected, indicated prevention).
- Study design; randomisation or details of control group selection in non‐randomised studies, duration of follow up, study duration, setting, blinding of participants and/or personnel.
- Outcomes measures; any information on the methodological quality of the measures used to measure primary or secondary outcomes. If measures are constructed or amended for a specific study details of this will also be extracted.
- Outcomes; Information relating to any of the primary or secondary outcomes including outcomes measured vs outcomes reported, who completed the measure. Timing of data collection including follow up data, drop‐out and completion rates. Outcome data will be extracted in all forms in which they are given (e.g. change data, endpoint data only, data for each category on ordinal scales).

##### Assessment of risk of bias in included studies

Two review authors will independently assess the risk of bias in included studies. For randomised and quasi‐randomised trials we will use the Cochrane Collaboration's 'Risk of bias' tool. For non‐randomised trials we will use a modified version of the Downs and Black methodological checklist for quality (Downs and Black, 1998). The results will be presented in a 'Risk of bias' table (Higgins, 2011).

For all included studies we will independently judge the included studies as having a low risk of bias ('Yes'), a high risk of bias ('No') or as having an uncertain risk of bias ('Unclear') and any uncertainty will be discussed with the review team. All information that led to a decision will be available. We will present our results using the following six domains:

##### Sequence generation

We will describe the method used in each study in enough detail so that a decision can be made on whether comparable groups were produced. We will judge the risk of bias as follows (see Higgins, 2011):

- 'Low' when a random component to generate the allocation sequence is described by the investigators e.g. throwing dice, coin tossing or using a computer random number generator.
- 'Unclear' when there is insufficient information to permit a judgement of 'Yes' or 'No'.
- 'High' when a non‐random component to generate the allocation sequence is described by the investigators e.g. the sequence is generated by date of birth/admission/date of intervention availability or by a judgement of the clinician or participant.

##### Allocation concealment

We will describe the method used to conceal allocation to determine whether the intervention allocation could have been identified in advance or during recruitment. We will judge the risk of bias as follows:

'Low' when assignment of the participants could not be foreseen because a one of the following, or equivalent, methods were used to conceal allocation: central allocation or sequentially numbered opaque sealed envelopes.

'Unclear' when there is insufficient information to permit a judgement of 'Yes' or 'No'.

'High' when assignment of the participants could have been foreseen which could have introduced selection bias e.g. using unsealed envelopes, a random number allocation schedule or any other unconcealed procedure.

##### Blinding

We will describe any measures used to blind study participants and personnel from knowing which intervention a participant may have received throughout the duration of the study. We will judge the risk of bias as follows:

- 'Low' when blinding of the participants and key personnel occurred, when the review authors are unlikely to be influenced by the lack of blinding or the outcome assessment was blinded.
- 'Unclear' when there is insufficient information to permit a judgement of 'Yes' or 'No' or the study did not address this outcome.
- 'High' when there was no or incomplete blinding of study personnel and participants (introducing bias or influencing the outcome measures) or when blinding could have been broken.

##### Incomplete outcome data

We will report data on attrition and exclusions (if included) from the analysis as well as the numbers involved (compared with those randomised). We will report reasons for attrition and exclusions where this is available and if any re‐inclusions were included in the analyses performed. We will judge the risk of bias as follows:

- 'Low' when there is no missing outcome data, when the reasons for missing data are unrelated to the outcomes, when missing data is balanced across both groups or when missing data have been imputed correctly.
- 'Unclear' when there is insufficient information to permit a judgement of 'Yes' or 'No,' when there is insufficient reporting of attrition and exclusions or the study did not address this outcome.
- 'High' when the reasons for missing data are likely to be related to the outcomes, when a substantial number of the intervention group have departed or when incorrect imputation procedures may have been followed.

##### Selective outcome reporting

We will assess the likelihood that the study authors overlooked some data when presenting the results by comparing the methodology with the results. We will judge the risk of bias as follows:

- 'Low' when the full study protocol is available and all primary and secondary outcomes have been reported in the pre‐specified way.
- 'Unclear' when there is insufficient information to permit a judgement of 'Yes' or 'No'.
- 'High' when not all of the study's primary (pre‐specified) outcomes have been reported, other methods than those pre‐specified were used, one or more outcomes are inadequately reported and cannot be used further (i.e., in a meta‐analysis) or the study fails to report results for a key outcome.

##### Other sources of bias

We will determine if any other bias was present in the studies by assessing whether there could have been any other problems not foreseen or acknowledged by the study authors. This may include preferential allocation, stopping the trial early or changing methods throughout the trial. We will judge the risk of bias as follows:

- 'Low' when the study seems to be free of other sources of bias.
- 'Unclear' when there is insufficient information to permit a judgement of 'Yes' or 'No' or insufficient rationale or evidence that an identified problem will introduce bias.
- 'High' when there is at least one other source of bias for example, a baseline imbalance, a biased study design was used or the study has been claimed to be fraudulent.

##### Risk of bias in non‐randomised studies

In addition to the above domains, non‐randomised studies risk of bias due to confounding (where a prognostic factor also predicts allocation to intervention) and bias in selection of participants into the study (where some eligible participants or outcomes from some participants are excluded) will be assessed using a modified version of the Downs and Black (1998) checklist for assessment of methodological quality (see Appendix C). This checklist consists of 34 questions to assess study reporting quality, external and internal validity (bias and confounding factors) and whether the study had sufficient power (Downs and Black, 1998). The checklist has been shown to have good test‐retest and inter‐rater reliability, along with high internal consistency. We will pay particular attention to studies which attempt to account for confounding through, for example, using a matched control group or controlling for relevant participant characteristics in the analysis. The main issue for clinical interventions will be that non‐randomisation means that perhaps more severe cases will be seen to have a greater need and are more likely to be allocated the intervention group, or on the other hand, if the researcher has a stake in the intervention they may be picky about who they accept (e.g., excluding severe externalising behaviours).

#### 4.3.6 Statistical procedures and conventions

##### Measures of intervention effect

Skewed data will be identified using the technique outlined in Chapter 9 of the Cochrane Handbook for Systematic Reviews of Intervention (maximum scale score minus the mean divided by the standard deviation (Higgins, 2011). A ratio of less than two indicates skew, while a ratio less than one indicates substantial skew). We will seek advice from a statistician if we identify substantial skew.

Dichotomous data will be analysed by calculating the odds ratio (OR) and its 95%confidence interval (CI). Continuous Data will be analysed by calculating the mean difference (MD) when outcomes are measured on the same scale or the standardised mean difference (SMD) when the same outcome is measured in different ways, with 95% CI. Ordinal Data will be pooled if authors are satisfied that studies are reporting data from instruments measuring the same underlying concept. If it is appropriate to pool data, we will either calculate MD or SMD or, if ordinal data is more usefully dichotomised for analysis data will be dichotomised and analysed as such. If it is not appropriate to pool findings we will present them narratively.

We will extract both change and endpoint data. For analysis, change data will be preferred over endpoint data. We will combine studies with change data in a meta‐analysis with studies with endpoint data using the (unstandardised) mean difference method. We will present MD change data in one subgroup, MD in endpoint data in another, and pool both subgroups for an overall analysis (Higgins, 2011). We will conduct sensitivity analysis to check the validity of this approach.

### 4.4 Unit of analysis issues

#### 4.4.1 Cluster randomised trials

School or centre based interventions are likely to use cluster‐randomisation, where randomisation occurs at a group level (e.g., classrooms or schools are randomised rather than individuals). For these trials we will adhere to the to the guidance on statistical methods for managing data from cluster‐randomised controlled trials provided by the Cochrane Handbook of Systematic Reviews of Interventions (Higgins, 2011, Section 16.3). We will check that adequate adjustments for clustering were made for estimates of intervention effects. If not, we will seek to extract or calculate effect estimates and their standard errors as for a parallel group trial, and adjust the standard errors to account for the clustering (Donner & Koval, 1980). This requires information on an appropriate intraclass correlation coefficient (ICC), which provides information on the relative variability in outcome within and between clusters (Donner & Koval, 1980). If this information is not available in the relevant report, we will request the information from the study authors. If this is not available or we receive no response, we will use external estimates obtained from studies that provide the best match on outcome measures and types of clusters from existing databases of ICCs (Ukoumunne, Gulliford, Chinn, Sterne & Burney, 1999) or other studies within the review. If we are unable to identify an appropriate ICC, we will perform sensitivity analyses using a high ICC of 0.1, a moderate ICC of 0.01 and a small ICC of 0.00. These values are rather arbitrary but, as it is unlikely that the ICC is actually 0, it is preferable to use them to adjust the effect estimates and their standards errors. We will combine the estimates and corrected standard errors from cluster randomised controlled trials with those from parallel designs using the inverse variance method.

### 4.5 Cross‐over trials

Should we identify any relevant studies in which participants received both the control and intervention but in a different order, we will only use the data collected up to the first crossover point of the study, in order to avoid any potential problems such as a carry‐over effect.

### 4.6 Dealing with missing data

If insufficient data has been reported to allow calculation of effect sizes, such as missing standard deviations or standard errors, we will contact authors in the first instance to obtain relevant data. Should we identify studies in which there has been substantial unaccounted for attrition from the study, we will still include these studies and describe the potential bias introduced by these studies and conduct a sensitivity analysis to explore the impact of studies with substantial loss to follow‐up on intervention effects. We will assess the sensitivity of any primary meta‐analyses to missing data using the strategy recommended by Higgins, White, and Wood (2008).

### 4.7 Assessment of heterogeneity

We will first assess clinical heterogeneity by considering the distribution of key participant factors (age, gender, severity of exposure, inclusion criteria) and trial factors (intervention mechanism as described by study authors, risk of bias due to confounding, selection bias, missing data). Should we identify any unexpected variability, we will discuss it in full. We will describe statistical heterogeneity by computing the I² statistic (Higgins & Thompson, 2002). In addition, we will conduct the *χ*² test of homogeneity to determine the strength of evidence regarding the genuineness of that heterogeneity. If heterogeneity is substantial (defined as an I² of at least 50%, and a *χ*² statistic with *p* value less than .10), we will explore reasons for heterogeneity (see Subgroup analysis and investigation of heterogeneity).We will also report between study variance in effect size using Tau².

### 4.8 Assessment of reporting biases

We will try to minimise the impact of reporting bias through thorough searching of both published and unpublished sources. Where there are a sufficient number of studies, usually considered to be a minimum of 10, we will assess the possibility of publication bias (on primary outcomes) using funnel plots of effect estimates on the horizontal axis against their standard errors (on the vertical axis on a reversed scale).We will apply Egger's regression asymmetry test to funnel plots to test for funnel plot asymmetry (Egger, 1997).

### 4.9 Data synthesis

We will synthesise universal and non‐universal interventions separately. This is due to the likely differences in the populations targeted. We considered synthesising the data by universal, selected and indicated interventions but decided against this because of the potential overlap between selected and indicated interventions. Studies may not make the distinction between selected or indicated interventions, or not be sufficiently clear in their aims or target participants to allow us to sensibly categorise interventions as either selected or indicated. We therefore propose to undertake the following comparisons for each outcome using a random effects meta‐analysis:

1. Universal interventions compared to control
2. Non‐universal interventions compared to control

Meta‐regression analysis will be undertaken to explore the influence of intervention components and study design. In this review we would like to adopt a different approach to allow randomised and non‐randomised studies to be analysed together: we are suggesting that we will pool the findings of randomised and high quality non‐randomised trials together.

We plan to use a random effects model as included studies are likely to be heterogeneous. In using a random effects model, study design can be controlled for by adding in an independent variable representing study design, randomised or non‐randomised. If this is coded so that the reference category (coded '0') is randomised studies then we can use the resultant meta‐regression model to generate unbiased effect size estimates for this reference category. The dummy variable will thus partition out the variation due to biases associated with non‐randomisation. We can also use it to assess whether, in this case, non‐randomised studies have introduced bias to the findings by considering its estimated coefficient and whether it is statistically significant. Only high quality non‐randomised studies will be included in this pooled analysis (those at low or moderate risk of bias). Non‐randomised studies that are judged to be at serious or critical risk of bias will not be included in the pooled analysis and instead be described narratively. If it is inappropriate to combine the results of any studies in a meta‐analysis, we will describe them narratively.

### 4.10 Subgroup analysis and investigation of heterogeneity

We will explore possible sources of heterogeneity using subgroup analyses based on:

- Gender (male only, female only, mixed groups);
- Age (infant, pre‐schooler, primary school age),
- Target of the intervention (children only, caregiver only, both children and caregivers),
- Location (low and middle income vs high income setting),
- Setting (ongoing vs post‐conflict setting).

### 4.11 Sensitivity analysis

We will conduct sensitivity analysis to explore the impact of procedural decisions and risk of bias. First, we will explore the impact of including both randomised and non‐randomised studies in meta‐regression. Second we will explore the impact of risk of bias by assessing the impact of removing studies judged to be at high risk of bias due to missing data/incomplete outcome reporting.

#### 4.11.1 Treatment of qualitative research

We do not plan to include qualitative research.

## ROLES AND RESPONSIBILITIES

Hanratty will have overall responsibility for the design, conduct and write up of the systematic review. The team will have regular meetings to coordinate progress and ensure that all members contribute to all aspects of the review. However, and within this, the particular expertise and lead contributions of team members will be as follows:

- Content: Hanratty, Neeson, Bosqui, Duffy.
- Systematic review methods: Hanratty, Neeson, Dunne.
- Statistical analysis: Connolly, Hanratty.
- Information retrieval (searching, screening and data extraction): Hanratty, Neeson, Bosqui, Dunne, Duffy.

## SOURCES OF SUPPORT

The Atlantic Philanthropies and the Bernard van Leer Foundation, UK provided funding for this systematic review through their support for Una: The Global Learning Initiative on Children and Ethnic Diversity. The funder has no input/influence on the design, conduct or reporting or this review.

## DECLARATIONS OF INTEREST

None of the review authors have a financial interest in this review. None of them have been involved in the development of interventions on the scope of the present one.

## PRELIMINARY TIMEFRAME

Approximate date for submission of the systematic review: December 2019.

## PLANS FOR UPDATING THE REVIEW

The lead author will hold responsibility for updating the review and commits to updating the review every 3 years. Should the author not be in a position to update the review within this timeframe the review can be updated by another team if needed.

## AUTHOR DECLARATION

### Authors' responsibilities

By completing this form, you accept responsibility for preparing, maintaining and updating the review in accordance with Campbell Collaboration policy. The Campbell Collaboration will provide as much support as possible to assist with the preparation of the review.

A draft review must be submitted to the relevant Coordinating Group within two years of protocol publication. If drafts are not submitted before the agreed deadlines, or if we are unable to contact you for an extended period, the relevant Coordinating Group has the right to de‐register the title or transfer the title to alternative authors. The Coordinating Group also has the right to de‐register or transfer the title if it does not meet the standards of the Coordinating Group and/or the Campbell Collaboration.

You accept responsibility for maintaining the review in light of new evidence, comments and criticisms, and other developments, and updating the review at least once every five years, or, if requested, transferring responsibility for maintaining the review to others as agreed with the Coordinating Group.

### Publication in the Campbell Library

The support of the Coordinating Group in preparing your review is conditional upon your agreement to publish the protocol, finished review, and subsequent updates in the Campbell Library. The Campbell Collaboration places no restrictions on publication of the findings of a Campbell systematic review in a more abbreviated form as a journal article either before or after the publication of the monograph version in Campbell Systematic Reviews. Some journals, however, have restrictions that preclude publication of findings that have been, or will be, reported elsewhere and authors considering publication in such a journal should be aware of possible conflict with publication of the monograph version in Campbell Systematic Reviews. Publication in a journal after publication or in press status in Campbell Systematic Reviews should acknowledge the Campbell version and include a citation to it. Note that systematic reviews published in Campbell Systematic Reviews and co‐registered with the Cochrane Collaboration may have additional requirements or restrictions for co‐publication. Review authors accept responsibility for meeting any co‐publication requirements.

## APPENDIX A

Database: Ovid MEDLINE(R) <1946 to present>

Search Strategy:

1 exp "warfare and armed conflicts"/

2 (war or wars or warfare or wartorn or war‐torn or warzone$ or post‐war$ or postwar$).tw,kw.

3 armed conflict$.tw,kw.

4 (conflict adj1 (area or zone$)).tw,kw.

5 (post‐conflict$ or postconflict$).tw,kw.

6 ((communal or violen$ or political or military) adj2 conflict$).tw,kw.

7 violence/

8 exposure to violence/

9 exp ethnic violence/

10 ((political$ or military or communal or state) adj2 violen$).tw,kw.

11 ((communal or communit$ or ethnic$ or military or political$ or state) adj2 violen$).tw,kw.

12 (ethnic cleansing or genocid$).tw,kw.

13 exp terrorism/

14 exp Civil Disorders/

15 (civil violence or civil conflict or civil disorder$ or riot$).tw,kw.

16 (terrorist$ or terrorism or insurgen$).tw,kw.

17 or/1–16

18 exp infant/

19 child/

20 (infan$ or baby or babies or toddler$ or child$ or boy* or girl* or preschool$ or pre‐school$ or kindergarten$ or kinder‐garten$ or preteen$ or pre‐teen$ or pre‐adolescen$ or schoolchild$ or student$ or juvenile$ or toddler$).tw,kw.

21 or/18–20

22 17 and 21

23 exp psychotherapy/

24 psychotherap$.tw,kw.

25 therapy.fs.

26 social support/

27 education/

28 exp mind‐body therapies/

29 Mental Health Services/

30 (Biofeedback or Breathing Exercises or hypnosis or Imagery or Meditation or Mindfulness or Psychodrama or Tai Ji or neurofeedback or Yoga or therapuetic touch).tw,kw.

31 (Aromatherapy or Bibliotherapy or Counsel?ing or Crisis Intervention or trauma‐focus$).tw,kw.

32 ((Animal or Art or colour or colour or dance or gestalt or laughter or music or narrative or person‐centred or child‐centred or play or reality or socioenvironmental) adj1 therap$).tw,kw.

33 ((Animal or Art or colour or colour or dance or gestalt or laughter or music or narrative or person‐centred or child‐centred or interpersonal or play or reality or socioenvironmental) adj1 therap$).tw,kw.

34 ((Eye Movement Desensitisation and Reprocessing) or EMDR).tw,kw.

35 (psychotherap$ or psycho‐therap$).tw,kw.

36 (psychosocial or psycho‐social).tw,kw.

37 ((cognit$ or behav$ or psychol$) adj3 (intervent$ or program$ or therap$ or treat$)).tw,kw.

38 or/23–37

39 22 and 38

## APPENDIX B DATA EXTRACTION FRAMEWORK

**Table:**

Study characteristics		
Report ID		
Study ID		
Type of report (Journal, Book, Conference, etc)		
Study Author		
Year of Publication		
Year of Study		
Journal/Source		
Contact Details		
Study Design		
Study Duration		
Ethical approval obtained from		
Consent procedures?		
Original Language of Report		
Protocol URL		
**Risk of Bias**	**Judgement (High, Low, Unclear)**	**Comments**
Sequence Generation		
Allocation Concealment		
Blinding of participants		
Blinding of outcome assessors		
Incomplete Outcome Data		
Selective Outcome Reporting		
Other Sources of Bias		
**Participant Characteristics**	**Intervention Group**	**Control Group**
Total No. of Participant Randomised		
Total No. of Participant Post Test		
Total No. of Participant At Final Follow up		
Total No. of Participants Analysed		
Age of Participants		
Gender of Participants		
How participants were recruited		
Geographical Location of Study		
Inclusion criteria		
Exclusion criteria		
**Characteristics and context of conflict**		
i.e. to highlight whether the conflict is ongoing or not, whether it is an international or civil war etc, to try to get a sense of the kind of violence the child is likely to have experienced or witnessed. This will help add context to our review.		
**Intervention Characterictics**		
Name of Intervention		
Mechanisms of change i.e. what components are in the intervention. For example, psychoeducation, providing safe spaces for children, support for parent‐child relationship, practical support such as providing food, shelter and clothing or providing support for families		
Type of Intervention i.e eg trauma processing, cognitive modification, attachment security, emotional regulation, education/insight etc		
Delivered to i.e. group or individual sessions?		
Location of intervention		
Developed by		
Design		
Duration		
Delivered when i.e. how soon after exposure was the intervention delivered?		
Delivered by?		
Other components?		
Limitations		
Cultural Adaptions		
Comparison/control		
**Measures**	**Scale**	**Summary**
**Outcome Data (baseline)**		
	**Intervention Group**	**Control Group**
Administered when?		
Sample characteristics		
**Analysis**		
Package		
Method		
**Outcome Data (1st follow up)**		
	**Intervention Group**	**Control Group**
Length of Follow‐up		
Summary		
Effect size?		
Limitations?		
Adverse effects		

## APPENDIX C CHECKLIST FOR MEASURING STUDY QUALITY (ADAPTED FROM DOWNS & BLACK, 1998; KENNELLY, 2011)

**Table:**

	YES	NO	Unclear
**Reporting**			
1. Is the hypothesis/aim/objective of the study clearly described?			
2. Are the main outcomes to be measured clearly described in the Introduction or Methods section?			
3. Are the methods of inquiry are appropriate for the study's aims?			
4. The authors discussed why they chose to use those methods?			
5. Are the characteristics of the patients included in the study clearly described?			
6. Are the interventions of interest clearly described?			
7. Are the distributions of principal confounders in each group of subjects to be compared clearly described?			
8. Are the main findings of the study clearly described?			
9. Does the study provide estimates of the random variability in the data for the main outcomes?			
10. Have all important adverse effects that may be a consequence of the intervention been reported?			
11. Have the characteristics of patients lost to follow‐up been described?			
12. Have actual probability values been reported for the main outcomes, except where the probability value is less than 0.001?			
**External Validity**			
13. Were the subjects asked to participate in the study representative of the entire population from which they were recruited?			
14. Were those subjects who were prepared to participate, representative of the entire population from which they were recruited?			
15. Were the staff, places, and facilities where the patients were treated, representative of the treatment the majority of patients received?			
**Internal Validity – bias**			
16. Was an attempt made to blind study subjects to the intervention they have received?			
17. Was an attempt made to blind those measuring the main outcomes of the intervention?			
18. If any of the results of the study were based on “data dredging,” was this made?			
19. In trials and cohort studies, do the analyses adjust for different lengths of follow‐up of patients, or in case control studies, is the time period between the intervention and outcome the same for cases and controls?			
20. Were the statistical tests used to assess the main outcomes appropriate?			
21. Was compliance with the intervention/s reliable?			
22. Were the main outcome measures used accurate (valid and reliable)?			
**Internal Validity – confounding (selection bias)**			
23. Were the patients in different intervention groups (trials and cohort studies) or were the cases and controls (case‐control studies) recruited from the same population?			
24. Were the study subjects in different intervention groups (trials and cohort studies) or were the cases and controls (case‐control studies) recruited over the same period of time?			
25. Were study subjects randomised to intervention groups?			
26. Was the randomised intervention assignment concealed from both patients and health care staff until recruitment was complete and irrevocable?			
27. Was there adequate adjustment for confounding in the analyses from which the main findings were drawn?			
28. Were the losses of patients to follow‐up taken into consideration?			
**Power**			
29. Did the study have sufficient power to detect a clinically important effect where the probability value for a difference being due to chance is 5%?			
**Research Value**			
30. Do the study findings contribute to the current knowledge base			
31. Can the findings reasonably be expected to inform current practices or polices?			
32. Are these contributions discussed by the authors?			
33. Have the authors identified new research areas?			
34. Have the authors discussed how the research findings could be used and for what populations?			
